# Effects of methylphenidate on the ERP amplitude in youth with ADHD: A double-blind placebo-controlled cross-over EEG study

**DOI:** 10.1371/journal.pone.0217383

**Published:** 2019-05-31

**Authors:** Mica Rubinson, Itai Horowitz, Jodie Naim-Feil, Doron Gothelf, Nava Levit-Binnun, Elisha Moses

**Affiliations:** 1 Department of Physics of Complex Systems, The Weizmann Institute of Science, Rehovot, Israel; 2 Beer Yaacov–Ness Ziona Mental Health Center, Beer Yaacov, Israel; 3 Faculty of Medicine, Tel Aviv University, Tel Aviv, Israel; 4 Child and Adolescent Psychiatry Unit, The Edmond and Lily Safra Children’s Hospital, Sheba Medical Center, Tel Hashomer, Israel; 5 Sagol Center for Brain and Mind, School of Psychology, Interdisciplinary Center, Herzliya, Israel; UNAM FES-Iztacala, MEXICO

## Abstract

Methylphenidate (MPH) is a first line drug for attention-deficit/hyperactivity disorder (ADHD), yet the neuronal mechanisms underlying the condition and the treatment are still not fully understood. Previous EEG studies on the effect of MPH in ADHD found changes in evoked response potential (ERP) components that were inconsistent between studies. These inconsistencies highlight the need for a well-designed study which includes multiple baseline sessions and controls for possible fatigue, learning effects and between-days variability. To this end, we employ a double-blind placebo-controlled cross-over study and explore the effect of MPH on the ERP response of subjects with ADHD during a Go/No-Go cognitive task. Our ERP analysis revealed significant differences in ADHD subjects between the placebo and MPH conditions in the frontal-parietal region at 250ms-400ms post stimulus (P3). Additionally, a decrease in the late 650ms-800ms ERP component (LC) is observed in frontal electrodes of ADHD subjects compared to controls. The standard deviation of response time of ADHD subjects was significantly smaller in the MPH condition compared to placebo and correlated with the increased P3 ERP response in the frontoparietal electrodes. We suggest that mental fatigue plays a role in the decrease of the P3 response in the placebo condition compared to pre-placebo, a phenomenon that is significant in ADHD subjects but not in controls, and which is interestingly rectified by MPH.

## Introduction

Attention-deficit/hyperactivity (ADHD) is a developmental psychiatric disorder involving problems with attention and/or hyperactivity and impulsivity. ADHD is often associated with anxiety, depression, difficulties with social interactions, learning disabilities and behavior disorders [1,2]. While the prevalence of ADHD is estimated to be higher than 10% among children [3], the neuronal causes of the disorder are still not fully understood. The standard of care medication in most cases is methylphenidate (MPH), which acts as a stimulant that intervenes in dopamine uptake. However, its role in curbing the disorder is also not fully elucidated yet.

Electroencephalography (EEG), an inexpensive and non-invasive method, enables the study of neurotransmission and is a means to understand neurobiological dysregulation [4]. Thus, it holds promise to enable identification of physiological biomarkers for diagnosis of ADHD [5] and the various effects of medications prescribed to alleviate its symptoms [6]. Specifically, time-locked EEG (event-related potentials) enables the investigation of sensory, perceptual, attentional and cognitive processes [4,7] with high temporal sensitivity. It is thus highly relevant in the investigation of how neural dysregulation in ADHD affects these processes and how their regulation is attained following medication [8]. Relevant event-related potentials (ERPs) include the minimum peak around 100ms (N1 component), which is considered to be an orienting or matching response that is observed when an unexpected stimulus is presented [9]; the maximum at about 200ms (P2) which is thought to relate to perceptual processing [10]; the minimum that closely follows P2, also in the vicinity of 200ms (N2), which is thought to be involved in inhibitory control [11]; the maximum around 300ms (P3), which is associated with the level of attention and with stimulus classification [12]; and the late components (>500ms) which relate to updating of working memory and retrieval from long-term memory [13].

Accordingly, extensive investigation of ERP differences between subjects with and without ADHD during various cognitive tasks, such as the Go/No-Go, oddball, Stroop, auditory and working-memory tasks, has been performed over the past years. This has identified differences mostly in the earlier components, namely N1, P2, N2 and P3 [14–16]. Some anecdotal evidence for differences between ADHD and controls was also found in later components (LC) such as the P500 [17].

The P3 component is often reported to be abnormally smaller in children with ADHD compared to controls during attention-related tasks, such as the continuous performance task (CPT) [18,19] and the Go/No-Go task [20]. In contrast, a study which examined adolescents with ADHD found no change in P3 during the Go/No-Go task [21]. Inconsistencies were also reported for the N2 component. On one hand, studies found reduced N2 in adolescents (25 boys and 2 girls) with ADHD during the Go/No-Go task [21] and in boys with ADHD during CPT [18] relative to controls. On the other hand, no change in N2 was detected in studies which examined mostly boys (27 of 28 participants) during the Go/No-Go task [20] or a mixed-gender group of children during CPT [19].

Furthermore, studies involving CPT found reduced N1 in boys with ADHD [18], yet larger N1 was found for mixed-gender children with ADHD [19] relative to controls. The first study also reported reduced P2, while the latter did not mention the P2 component. Other studies involving the Go/No-Go task [20,21] did not report any change in N1 or P2. These inconsistencies may be attributed to a range of parameters that differed across studies, including differences in tasks and task difficulty, subjects’ age and gender, diagnostic criteria, symptom severity and comorbidity with other conditions, use of different incentives and variability in data analysis techniques (Barry et al., 2003 [22] for a review and discussion of these inconsistencies).

In addition to inconsistencies between studies, a gap exists in the analysis of previous work, since most of it has ignored the later components (>500ms post stimulus), focusing instead on the earlier components. However, late components do provide important information, for example, De France et al., 1996 [17] suggested the P500 component as an effective discriminator between controls and two sub-groups of ADHD and Slopen at al., 2012 [23] suggested that disruptions in the P700 component may be linked to ADHD. Therefore, in this study we analysed the ERP signals over a wide time window (0-1000ms post stimulus), to account for both the earlier and the later components.

Methylphenidate (MPH) is the most prescribed medication for ADHD [24] and is known to help with increasing and maintaining alertness, combating fatigue, and improving attention. Improvements in cognition, including working and episodic memory, and in inhibitory control, were also reported [25]. While MPH is considered an effective stimulant [26], the neuronal reasons for its high efficacy are still unclear. Several EEG studies have attempted to address this question by comparing the ERPs of subjects with ADHD after treatment with MPH to those who received a placebo treatment. Unfortunately, findings on amplitudes and latencies of N1, N2, P2 and P3 components are yet again inconsistent across studies.

For example, several studies involving CPT (X version) reported mixed-gender children with ADHD having abnormally small P3 amplitudes compared to controls, which were normalized after administration of MPH [27–30]. However, other studies on similar subjects found no difference in the P3 amplitude in response to MPH during auditory stimuli [31] or during CPT (double version) [32]. The latter study, along with other studies which involved the visual oddball task [14,33], revealed longer P3 latencies in mixed-gender children with ADHD relative to controls, which were normalized after MPH intake. However, no changes were evident in the P3 latency between responders and non-responders to MPH during an auditory oddball task [14]. The N2 amplitude of mixed-gender children with ADHD was found to increase after MPH intake during CPT [27], yet other CPT studies on boys of similar age did not report any N2 changes following MPH [18,34]. Similarly, the N1 amplitude was found to increase in boys with ADHD during CPT following MPH intake [18,34] but was not reported in another study on mixed-gender children with ADHD [27]. No P2 changes following MPH were reported in these studies [18,27,34].

In addition to the reasons mentioned above that may lead to variability between studies, a main reason for inconsistencies across these MPH studies has been attributed to the varied experimental designs [15]. Due to the variability of the ADHD symptoms across subjects, there is great importance for a within-subjects design. Despite this, only a few EEG studies used a within-subject design [27,28,32,34]. For example, in a double-blind placebo-controlled cross-over design, Verbaten et al., 1994 [27] and Klorman et al., 1979 [28] compared MPH versus placebo effects during a CPT task and found increased P3 amplitude following MPH. Yet, both studies administrated the treatments on separate days without a preceding treatment-free session, and therefore could not account for between-days variability. In another study, Klorman et al., 1999 [34] examined the ERP response of ADHD subjects during an auditory oddball cognitive task after a counter-balanced treatment of 14-days and found an increase in the N1 amplitude after MPH ingestion compared to placebo. As this study included only one drug-free baseline session which was performed in the beginning of the protocol and was not repeated for every test day, it also could not account for between-days variability. Sunohara et al., 1999 [32] built on these studies and employed a design in which the effect of different MPH dosages on the ERP response was tested four times in two days (two sessions in each day) during a CPT (double version). In this design, the first session served as baseline while the other three sessions enabled to test the effects of low and high doses of MPH relative to placebo. Controls in this study were assessed once. Surprisingly, the ERP amplitudes were unaffected across the experimental conditions. However, changes in ERP latencies were found. Specifically, the ADHD group at baseline had shorter P2 and N2 latencies and longer P3 latencies compared to controls. Furthermore, higher dose of MPH was associated with increased P2 and N2 latencies and decreased P3 latencies. Since this study included only one drug-free baseline session, it could not account for potential baseline variability [35,36], for any learning effects from repeating the task [37] or for mental fatigue [38].

To overcome some of these limitations, and building upon Sunohara et al., 1999 [32], we conducted a double-blind placebo-controlled cross-over design using the SART (Go/No-Go) task [39]. ADHD subjects were required to participate in two test days during which they performed a baseline cognitive task in the morning and then randomly received either placebo or high dose MPH in the afternoon, which was followed by a repeat of the cognitive task. Control participants were required to participate in one test day and complete two consecutive cognitive sessions. Both early and late ERP components were inspected in our study. Our design enabled us to simultaneously: (1) investigate the differences in ERPs between ADHD and controls by comparing the first session of the first day between groups; (2) examine the normalization effect of MPH on ERPs by comparing the MPH session to its preceding baseline and to placebo, and by comparing the change following MPH to the healthy condition; (3) investigate the possible moderating effect of learning on the ERPs by comparing the two pre-treatment sessions, i.e., first session on the first day versus the first session on the second day; and (4) explore the moderating effect of fatigue on the ERPs by comparing the placebo session with its pre-treatment session.

As the SART task has been found to affect the P3 amplitude [20,40] and latency [32] of children with ADHD relative to controls, and MPH was found to normalize such effects in similar CPT tasks [27–30,32], we hypothesized that differences in the amplitude and latency of the P3 component would emerge between ADHD participants and controls, as well as effects of MPH versus placebo. Specifically, we expected smaller P3 amplitude and longer P3 latency in ADHD relative to controls. MPH was expected to increase the P3 amplitude and shorten the P3 latency relative to placebo. Furthermore, mental fatigue was previously shown to decrease the P3 amplitude [41], and therefore we anticipated differences in the P3 amplitude between two sessions of the same testing day. As the SART was shown to not result in learning effects [42], we did not expect any between-days variability.

## Materials and methods

### Participants

Twenty children diagnosed with ADHD and twenty-one gender- and age- matched controls were recruited for the study. The children with ADHD were recruited from the child and adolescent psychiatry unit in Sheba Medical Center in Tel Hashomer, Israel. Healthy controls were recruited through ads sent to the workers of the Weizmann Institute or posted on bulletin boards in the Interdisciplinary Center (IDC) in Herzliya, Israel.

One child with ADHD and two controls were excluded due to excessive noise in their EEG recordings. One control participant was excluded from the study due to technical errors during his EEG recordings. We therefore report results from a group of 19 ADHD participants (10 males; mean age = 12.1; SD = 2.5; age range = 9–17), and a comparison control group of 18 controls (10 males; mean age = 12.2; SD = 2.8; age range = 9–17).

Subjects with ADHD were evaluated by a trained psychiatrist during the first baseline visit, and were included in the study only if they were diagnosed with ADHD according to the Diagnostic and Statistical Manual of Mental Disorders (DSM5) criteria. Thirteen of the patients were naive to ADHD medications, and six were regular users of MPH. All participants with ADHD were diagnosed with at least a moderate severity of ADHD symptoms (Clinical Global Impression–Severity [CGI-S] score ≥ 4). All controls were screened by a child psychiatrist using the child strength and difficulties questionnaire and were not included in the study if they had a history of a psychiatric disorder or neurological cognitive impairment [43].

Criteria for exclusion from the ADHD study group were: age lower than 9 or above 18 years; history of seizure, head trauma, substance abuse or medication other than MPH; or an inability to swallow pills. Criteria for exclusion from the control group were: age lower than 9 or above 18 years; history of seizure, head trauma, substance abuse; current medication use; or current or lifetime psychiatric disorders.

The study was approved by the institutional review board at Sheba Hospital and was conducted according to the principles of the declaration of Helsinki. Since participants were children and adolescents, both parents signed an informed consent form and assent to participate in the study was obtained from all children. Subjects' parents were reimbursed for travel and loss of time.

### Experimental design, tasks and EEG recordings

#### Assessment by questionnaires

All participants (ADHD and controls) completed a general demographic questionnaire and a standardized intelligence test based on Raven's Progressive Matrices scale [44]. Subjects also completed the Positive Affect Negative Affect Scale (PANAS) [45] at the end of each testing session, in order to control for possible effects of mood on cognitive performance. Subjects with ADHD were further assessed by the Hebrew version of the Schedule for Affective Disorders and Schizophrenia for School-aged Children, Present and Lifetime [46]. Their parents and teachers also filled the Dupaul ADHD rating scale [47]. Demographic and clinical data are detailed in Table 1.

**Table 1:** Demographic and clinical data for subjects with ADHD and healthy control subjects (N/A- not applicable).

	ADHD	Controls
N	19	18
Gender (M:F)	10:9	10:8
Age (Mean ± SD)	12.1 ± 2.5	12.2 ± 2.8
Raven's adjusted score (Mean ± SD)	135± 15.9	135.5 ± 13.5
Socioeconomic status (high : medium : low)	16:3:0	16:2:0
Treated for comorbid disorders	1(enuresis)	0
Father’s education (years; Mean ± SD)	14.93 ± 2.35	17.46 ± 2.98
Mother’s education (years; Mean ± SD)	17.66 ± 3.06	17.15 ± 2.51
Dupaul parent ADHD rating scale (Mean ± SD)	49.2 ± 9.16	N/A
Dupaul teacher ADHD rating scale (Mean ± SD)	39.7 ± 12.6	N/A
Inattentive specifier (n)	8	N/A
Hyperactive specifier (n)	0	N/A
Combined specifier (n)	11	N/A
Clinical Global Index (CGI) (Mean ± SD)	4.68 ± 1.3	N/A

#### Procedure

For all participants, each experimental session consisted of the following steps:

1. A baseline EEG recording of 5 minutes, during which subjects were instructed to sit comfortably with their eyes open.
2. EEG recordings during which participants completed the computerized SART task [39]. Following the SART task, the participants also completed the n-back task and the Stroop color and word task. However, here we report on the data from the SART task only.

The number of sessions differed between the ADHD and control groups, as detailed below.

For the ADHD group, the study was performed based on a double-blind placebo-controlled cross-over design [48]. The subjects participated in 4 experimental sessions that were divided into two test days. On the first test day, subjects first completed the questionnaires listed above and then completed the first session with a BioSemi head-cap on their head (BioSemi, Amsterdam, The Netherlands). Then, 10 participants received MPH immediate release (IR) and 9 received placebo in a randomized manner (the dosage of MPH-IR was 0.5 mg/kg or 20 mg, whichever was smaller). The subjects were given an hour break during which the EEG cap remained on their head, before going on to complete the second session of day 1. The subjects were called back one week later for test day 2, in which they performed a third and fourth session, separated by an hour break in a similar manner. The subjects who received MPH IR on test day 1 received placebo on test day 2, and vice versa.

The controls performed two sessions on one test day and did not receive any medication. They were similarly given an hour break between the two sessions, during which the EEG cap remained on their head.

#### The SART task

Single digits (‘1’ through ‘9’) were presented in black centered on a white background. Subjects were asked to respond by pressing a designated button (Go condition), with the exception of the digit ‘3’, for which participants were asked to refrain from pressing (No-Go condition) [39]. A total of 297 trials (264 Go and 33 No-Go) were presented in a mixed and randomized order. The duration of each trial was 150 milliseconds, followed by an inter-stimulus interval (ISI) which varied between 1.5, 2 and 2.5 seconds. The variability of the ISI duration aims to minimize speed/accuracy tradeoffs [49]. A demonstration sequence of 18 trials (16 Go trials and 2 No-Go trials) was presented to the subjects following the baseline measurement but before the session itself. Participants were instructed to give equal importance to the speed and accuracy of their responses. The subjects’ performance was assessed by their response time (RT, the time between presentation of the digit on the screen and the pressing of the key) and by the amount of errors in commission and in omission.

#### Experimental set up

At the beginning of the test day, a BioSemi head-cap of suitable size (BioSemi, Amsterdam, The Netherlands) was placed on the participants’ head. Continuous EEG recordings were collected from 64 Ag-AgCl active electrodes (10/20 international system) at a sampling rate of 1024Hz.

For 9 ADHD subjects and 3 controls, some electrodes were removed due to excessive noise (a maximum of 6 electrodes, on average 3 electrodes, mostly in the parietal area). Out of the removed electrodes, only two (P9, which was removed in one ADHD subject, and PO4, which was removed in a different ADHD subject) are included in the reported results. For these electrodes, the data were reported from 18 subjects instead of 19.

For the ADHD group, the experiment took place at Sheba Hospital, Ramat Gan, Israel. Cognitive stimuli were presented to the subjects using the E-Prime software (version 2, Psychology Software Tools, Pittsburgh, PA). Triggers that marked the time of the stimuli presentation were sent from the E-prime software to the BioSemi system via a USB relay (KMTronic USB Relay; KMTronic, Veliko Turnovo, Bulgaria). For the control group, 9 subjects were also tested at Sheba Hospital with the same experimental system as the ADHD subjects. The other 9 subjects were tested at the Interdisciplinary Center (IDC), Herzliya, Israel. In this case, the E-Prime software sent triggers to the BioSemi system via a standard parallel port. Due to differences in the internal processing time between the systems in IDC and Sheba hospital, the experimental system at Sheba Hospital was 25ms faster than the experimental system at IDC and the data taken at IDC were corrected accordingly.

#### EEG pre-processing steps

The continuous EEG recordings were filtered in EEGLAB using a hamming-windowed 3^rd^ order linear Finite Impulse Response (FIR) band-pass filter (low cutoff at 1Hz, high cutoff at 80Hz (-6dB point), low transition bandwidth 0.2Hz, high transition band width 10Hz) and a notch-filter of 50Hz (low cutoff at 49Hz, high cutoff at 51Hz (-6dB point), transition bandwidth 8Hz). Go and No-Go data were segmented into epochs from -1000ms to 2000ms around the stimuli presentation, and an offset (calculated as the average of the signal from -350ms to 0ms) was removed for each epoch. Epochs which were excessively corrupted by movement artifacts were manually removed (about 50 in total) and the data were then referenced by subtracting the average of all electrodes [50]. To remove eye-blinks, lateral eye movements and auditory artifacts, Independent Component Analysis (ICA) was conducted and manually inspected using EEGLAB [51] to remove the three ICA components that contributed most to these artifacts. All trials with an error in the subjects’ response across the Go (ADHD: 2.85% of trials, controls: 1.97% of trials) and No-Go (ADHD: 43.7% of trials, controls: 31.3% of trials) conditions were removed and the analysis was performed only on the correct trials. Trials were averaged for each electrode to retrieve a mean response waveform (MRW) per subject. As very few correct No-Go trials were left after the cleaning process, we report results only from the correct Go trials. The 64 electrodes were assigned into 14 groups (centered in the right/middle/left frontal, central, parietal, occipital areas, and right/left temporal areas) based on the division detailed in Naim-Feil et al., 2018 [52]. This division is illustrated in S1 Fig. For every subject, the MRW were averaged over the electrodes of each group.

#### Statistical analysis

The statistical analysis was performed in Matlab (The MathWorks, Inc, Natick, MA). For each subject and group of electrodes, the MRW was divided into sliding windows of 150ms, which is the typical width of the ERP components. The windows were bounded between 0ms-1000ms post stimulus (with an overlap of 125ms between two consecutive windows) and the mean amplitude of the signal, averaged over time for the duration of the window, was calculated. Repeated measures ANOVA was performed for each window and group of electrodes. The statistical significance level α for all analyses was set by *p*≤0.05, and we considered significance only if it occurred in three or more consecutive windows.

For the ADHD group, three binary criteria describe each EEG recording: whether the recording preceded or followed the treatment (labeled “pre” or “post”); whether placebo or MPH was administrated (labeled “PLB” or “MPH”); and whether MPH was given on the first or second day (labeled “first” or “second”). We grouped the data into 4 conditions, “pre-PLB”,”pre-MPH”,”post-PLB” and “post-MPH”, and tested for differences between the groups using Repeated Measures ANOVA with Time (pre or post) and Intervention (PLB or MPH) as within-subject factors, and Order (first or second) as between-subjects factors. The subjects were classified as children (< = 12) or adolescence (>12) [22], and their age, gender and sub-type of disorder (inattentive or combined specifier) were considered as covariates. Post-hoc analyses with paired-sample t-tests were conducted for electrodes’ groups in which a significant “Time x Intervention” interaction was found.

To investigate the differences between ADHD and controls, we grouped the data of the ADHD subjects according to session number, and considered their two treatment-free sessions: the first session of the first day (labelled “S1”) and the first session of the second day, which was the third session overall (labelled “S3”). The control group performed two sessions on the same day and did not receive any treatment, and their sessions were simply labelled H1 (first session) and H2 (second session). Differences between the groups were tested using Repeated Measures ANOVA with Session (“first” or “second”) as a within-subject factor and Diagnosis (“control” or “ADHD”) as a between-subjects factor. Post-hoc analysis with unpaired-sample t-tests was conducted for electrodes’ groups in which a significant effect of “Diagnosis” was found.

To examine within-day changes, difference waves were calculated for each subject and group of electrodes. We subtracted the ERPs of the pre-placebo condition from the post-placebo condition (labelled as “ΔPlacebo”); the ERPs of the pre-MPH condition from the post-MPH condition (labelled as “ΔMPH”); and the ERPs of the H2 condition from the H1 condition (labelled as “ΔControl”). Then we could examine differences between the Δ conditions using paired t-tests.

Correlations between behavioral variables (performance variability, commission errors, omission errors and clinical assessment scores) and ERP amplitudes were calculated using the Pearson correlation coefficient. We considered those behavioral variables for which significant difference between groups were found. Furthermore, in order to avoid multiple comparisons errors during the correlation analyses, we focused only on time windows and electrodes’ groups in which significant differences in amplitude were already evident. For the relevant electrodes’ groups, the correlation analysis was performed per-electrode.

## Results

### Healthy controls vs. ADHD and testing for learning effects

**Fig 1:**
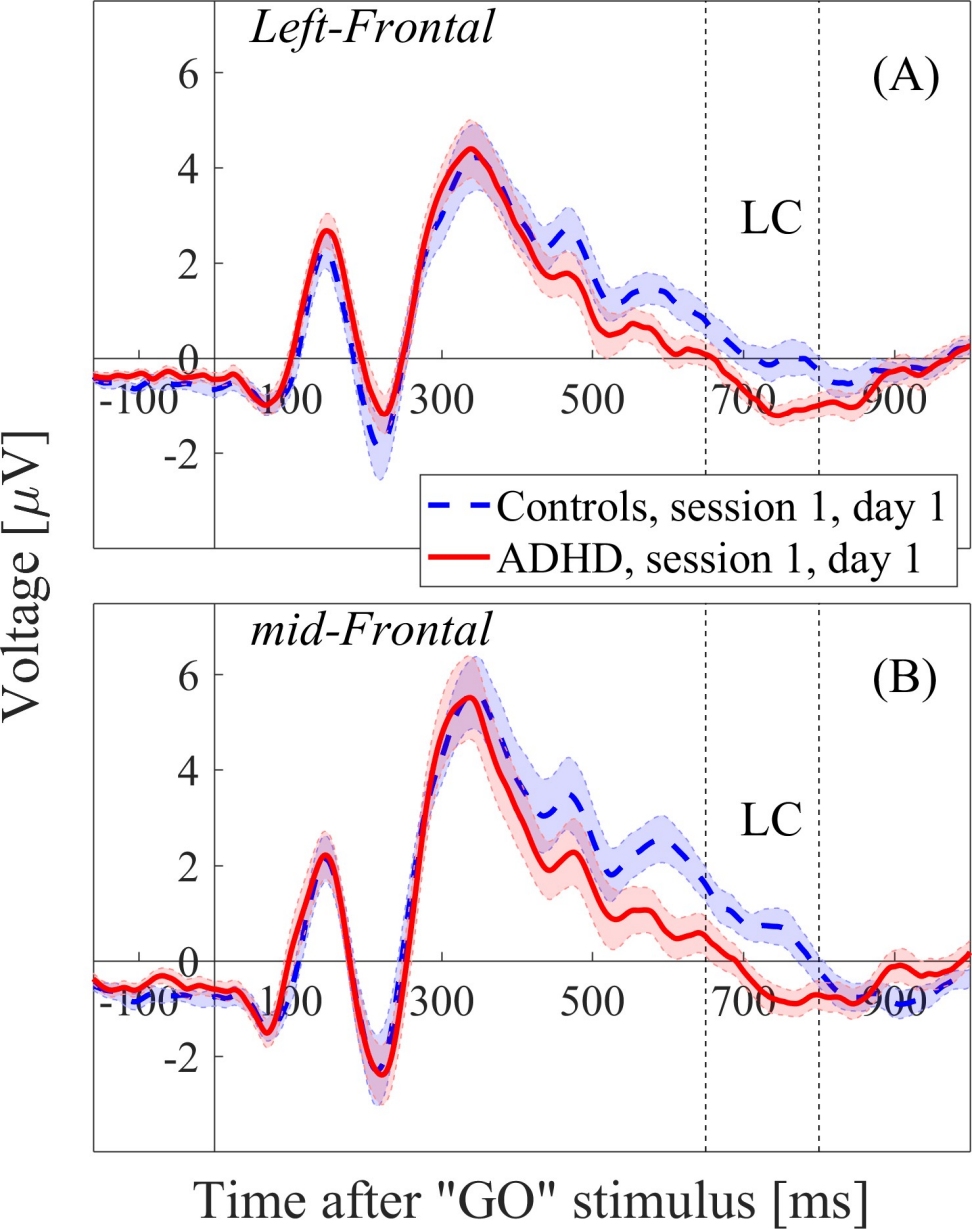
ERP waveforms of the first sessions of the Controls and ADHD subjects (H1 and S1, respectively) in the left frontal region (A) and middle frontal region (B). The LC window (650-800ms) is marked by dashed lines.

Differences between the groups were tested using Repeated Measures ANOVA with Session (“first” or “second”) as a within-subject factor and Diagnosis (“control” or “ADHD”) as a between-subjects factor. Significant “Diagnosis” effects (p < .05) were found in the left and middle frontal regions in 5 consecutive time windows, ranging from 550ms to 800ms post-stimulus. Post-hoc analysis revealed significant differences between the first session of the healthy controls (H1) and the first session of the ADHD subjects (S1). The statistical results are reported for the window 650-800ms, in which the effect-sizes (Cohen’s d) were most dominant (Left frontal: *F*_1,34_ = 5.5, *p* = 0.02, Cohen’s d = 0.89; Middle frontal: *F*_1,34_ = 8.2, *p* = 0.007, Cohen’s d = 0.94) (Fig 1). No “Session” effect or “Session x Diagnosis” interaction was found in these left-middle frontal regions.

### Placebo vs. MPH and fatigue effects

**Fig 2:**
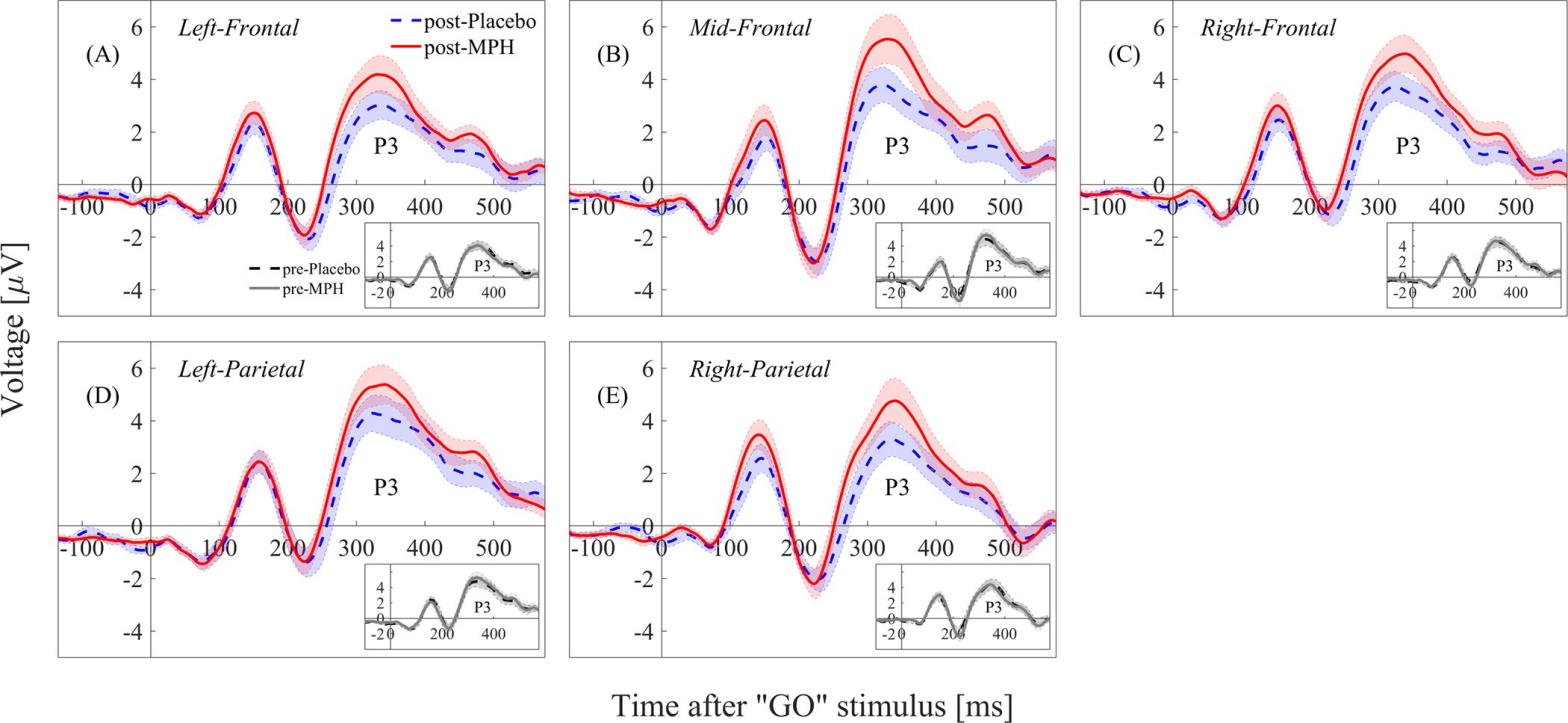
ERP waveforms of the post-placebo vs. post-MPH conditions (large panel) and pre-placebo vs. pre-MPH conditions (small panel). The waveforms are plotted for the right/left/middle frontal regions (panels A/B/C) and for the right/left parietal regions (panels D/E). A significant amplitude difference is evident between the post-placebo and post-MPH conditions in the P3 (250-400ms) component. MPH = methylphenidate.

Differences between the groups were tested using Repeated Measures ANOVA with Time (pre or post) and Intervention (PLB or MPH) as within-subject factors, and Order (first or second) as a between-subjects factor. Significant “Time x Intervention” effect (p < .05) was found in 4 consecutive windows ranging from 200ms to 500ms in the right/middle/left frontal regions and in the left/right parietal regions. No “Order” interaction was found, nor any age or gender effects. Post-hoc analysis revealed significant differences between the post-placebo and the post-MPH conditions, as well as between the pre-placebo and post-placebo conditions. The statistical results are reported for the third window, 250-400ms, in which the effect-sizes (Cohen’s d) were most dominant (Table 2, Fig 2). In both cases, the post-placebo condition had significantly lower amplitude in the relevant brain regions. No differences were found between the pre-MPH and post-MPH conditions.

**Table 2:** Means and standard deviations (SD) of the P3 component (250-400ms post stimulus) amplitudes in the relevant brain regions for pre/post-PLB and pre/post-MPH conditions, and post-hoc tests of significant differences in medication effects between the groups. Significance is marked by * (* p < .05, ** p < .01, *** p < .001). PLB = placebo, MPH = methylphenidate.

Region	PLB (n = 19)		MPH (n = 19)		Post-PLB vs. Post-MPH		
	Pre-PLB Mean (SD)	Post-PLB Mean (SD)	Pre-MPH Mean (SD)	Post-MPH Mean (SD)	*F*_*1*,*18*_	*p-Value*	Cohen’s d
**Right frontal**	3.21(2.05)	2.08(1.94)	2.93(2.03)	2.84(1.61)	18.14	0.0004***	0.97
**Middle frontal**	3.04(2.58)	1.66(2.24)	2.81(2.48)	2.81(2.11)	19.45	0.0003***	1.01
**Left frontal**	2.29(1.94)	1.40(1.87)	2.40(1.68)	2.18(1.19)	29.35	0.00003***	1.18
**Right parietal**	2.60(2.29)	1.53(1.92)	2.50(2.07)	2.25(1.51)	15.08	0.001***	0.99
**Left parietal**	2.93(2.04)	2.03(1.93)	2.65(1.86)	2.77(1.93)	10.92	0.004**	0.81

### Normalization effect of MPH

For the frontal and parietal regions that were found in Figs 1 and 2, we examined the conditions ΔPlacebo (post-placebo minus pre-placebo), ΔMPH (post-MPH minus pre-MPH) and ΔControl (difference between the two control sessions, H2 minus H1) for the 0-1000ms post-stimulus window.

**Fig 3:**
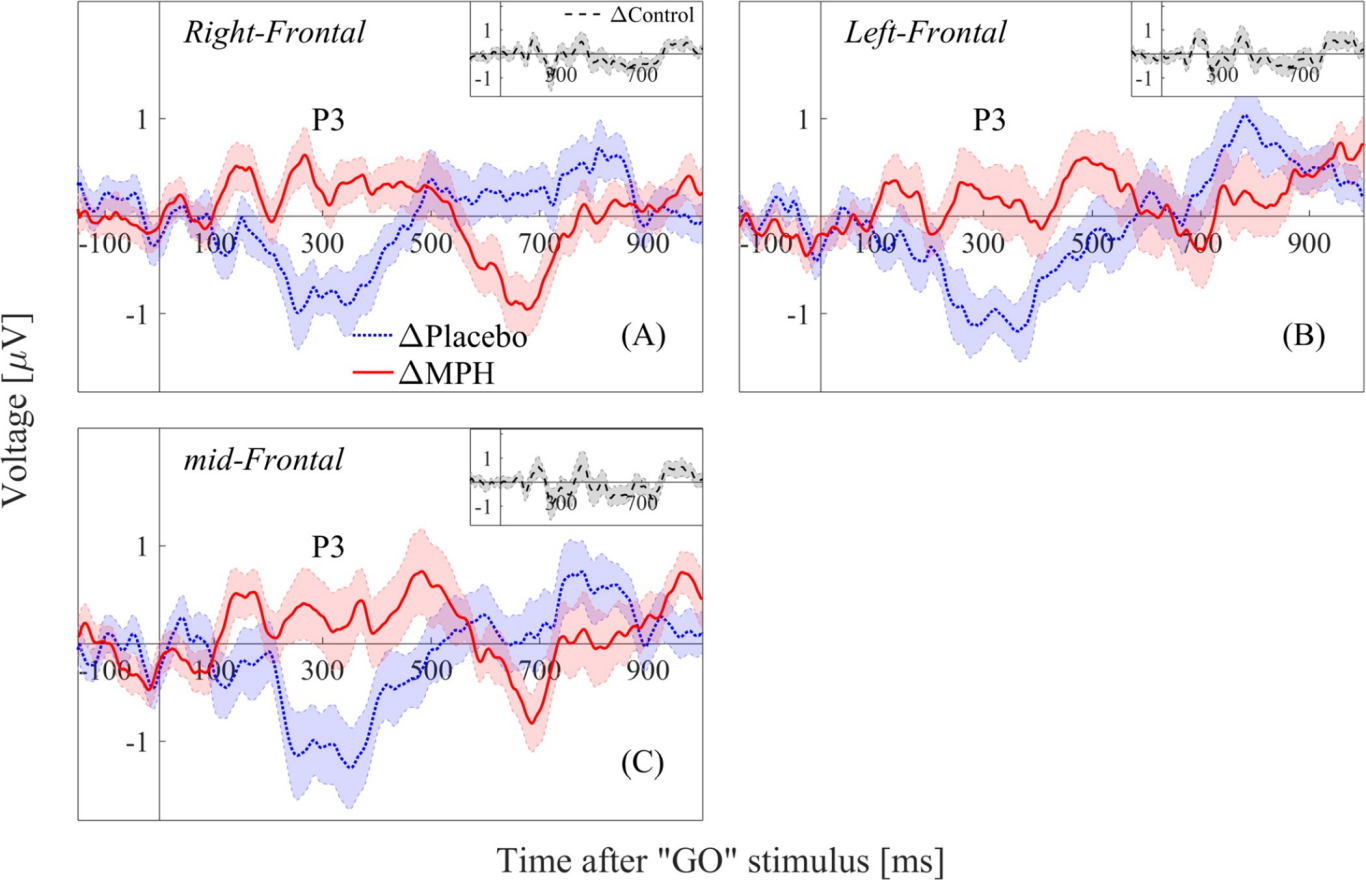
ΔPlacebo (post-placebo minus pre-placebo) vs. ΔMPH (post-MPH minus pre-MPH) (large panel) and ΔControl (H2 minus H1) (small panel) conditions for (A) right, (B) middle and (C) left frontal regions. MPH = methylphenidate.

A significant difference in the P3 amplitude (250-400ms) was evident in the right/middle/left frontal regions when comparing ΔPlacebo and ΔMPH (Left frontal: *F*_1,18_ = 10.35, *p* = 0.005, Cohen’s d = 0.72; Middle frontal: *F*_1,18_ = 13.02, *p* = 0.002, Cohen’s d = 0.83; Right frontal: *F*_1,18_ = 18.26, *p* = 0.0004, Cohen’s d = 0.98). Furthermore, a significant decrease in the amplitude of the ΔPlacebo condition was found in the P3 window (Left frontal: *F*_1,18_ = 6.36, *p* = 0.02, Cohen’s d = 0.59; Middle frontal: *F*_1,18_ = 9.37, *p* = 0.006, Cohen’s d = 0.71; Right frontal: *F*_1,18_ = 9.99, *p* = 0.005, Cohen’s d = 0.72), while no change was found in the ΔMPH condition. No significant change was evident in these regions for ΔControl. Finally, one can see that the waveform pattern of the ΔMPH condition follows the ΔControl waveform pattern along the full post-stimulus time window, suggesting a normalizing effect of the MPH towards the control’s brain activity (Fig 3).

### Correlations of EEG data to performance

Task performance results (Table 3) showed that ADHD subjects made significantly less commission errors post-MPH compared to pre-MPH (*p* = 0.005), while no difference was found post-placebo compared to pre-placebo. In addition, the ADHD subjects made significantly more omission errors post-placebo compared to pre-placebo (*p* = 0.01), but not for post-MPH compared to pre-MPH. No differences were found between the H1 and H2 conditions.

**Table 3:** Performance in the Go/No-Go task. Each entry represents the average over subjects followed by the variance in parentheses. Data for the mean response time (<RT>) and standard deviation of the response time (RTSD) relate only to the Go condition. H1 and H2 stand for the first and second sessions of the controls, respectively. PLB = placebo, MPH = methylphenidate.

Performance	ADHD (N = 19)				Controls (N = 18)	
	pre-PLB	post-PLB	pre-MPH	post-MPH	H1	H2
Commission errors	10.6(8.2)	11.3(7.9)	14.9(7.3)	10.9(7.6)	11.2(6.2)	8.1(4.4)
Omission errors	9.7(10.8)	19.4(21.2)	10.5(14.6)	5.2(9.5)	4.8(5.4)	9.6(13.9)
<RT> [ms]	471.7(124.5)	473.2(124.0)	447.7(97.7)	436.2(87.7)	456.9(87.2)	453.3(91.3)
RTSD [ms]	107.3(36.3)	123.9(40.5)	116.2(41.6)	87.1(32.8)	113.1(27.9)	116.2(40.1)

**Fig 4:**
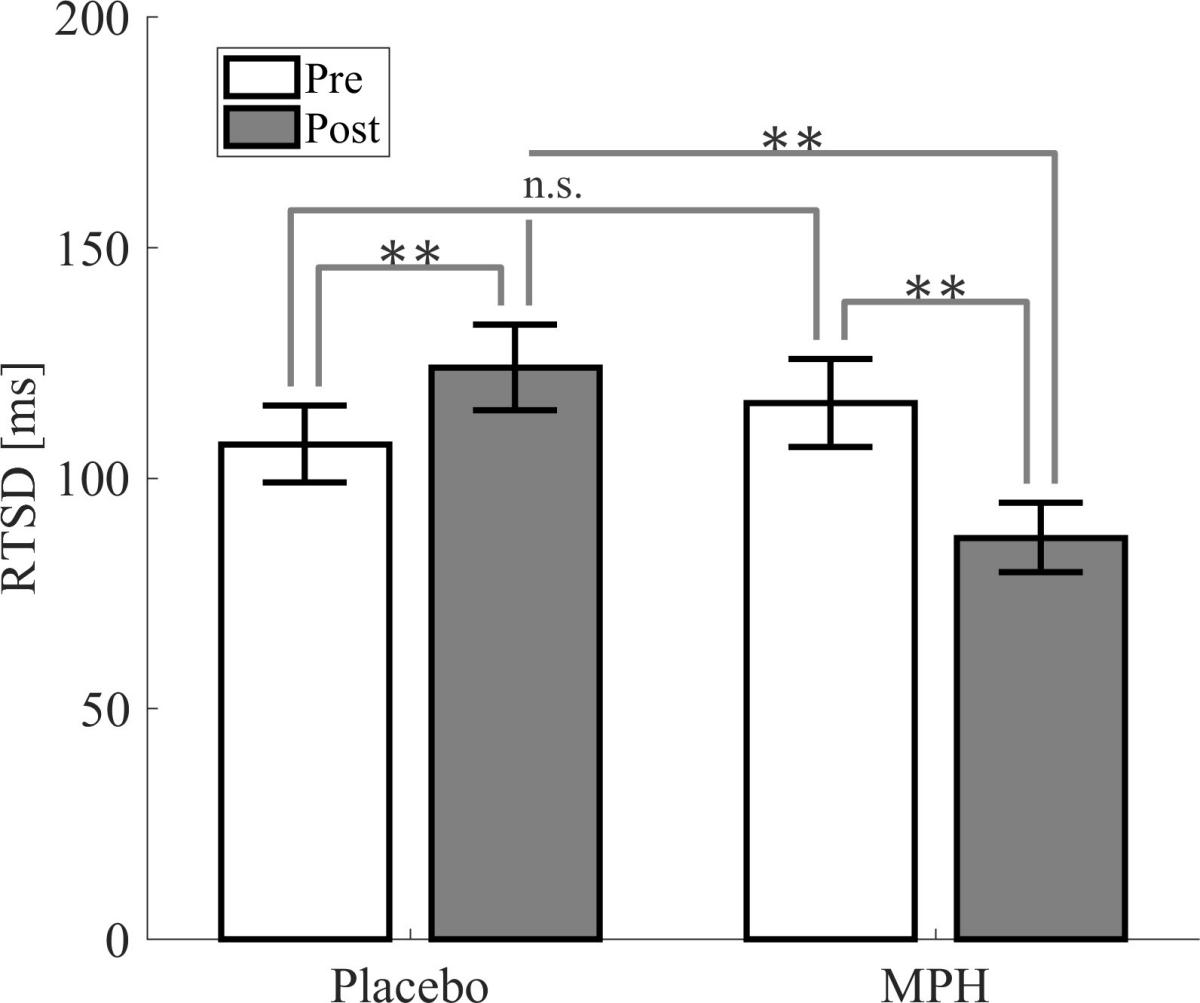
Standard deviation of the ADHD subjects’ response time (RTSD) in the Go condition for pre-placebo, post-placebo, pre-MPH and post-MPH conditions. Significant differences are marked by * (* *p* < .05, ** *p* < .01, *** *p* < .001). MPH = methylphenidate.

**Fig 5:**
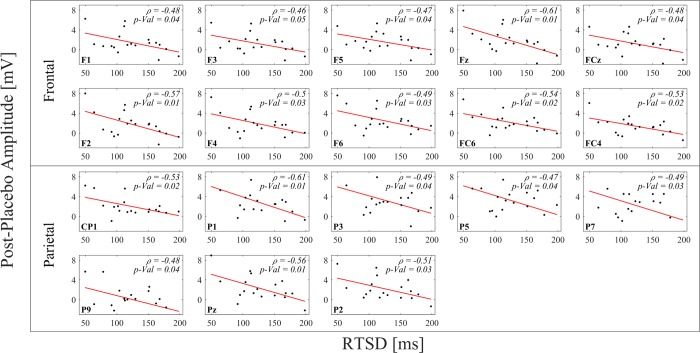
Pearson correlations between the amplitude of the post-placebo condition in the P3 component (250-400ms) and the standard deviation of the response time (RTSD). In each sub-plot, the data-points represent the 19 subjects with ADHD.

The standard deviation of the response time (RTSD) significantly differed across the conditions of subjects with ADHD (post-placebo>post-MPH, *p* = 0.001, see Fig 4), and was also found to correlate (*p*<0.05) to the P3 amplitudes (250-400ms) of the post-placebo condition in the parietal and frontal electrodes (F1, F3, F5, CP1, P1, P3, P5, P7, P9, Pz, Fz, F2, F4, F6, FC6, FC4, FCz and P2) (Fig 5).

## Discussion

Well-designed experiments are particularly important when inconsistency is reported in the literature, as is the case of ERP measured in ADHD with relation to cognitive tasks and to the effects of MPH. Less than a handful of studies have used placebo-controlled cross-over designs [27,28,32,34] which account for within-subject variability and placebo effects. Furthermore, none have accounted for between-days variability by performing a cognitive baseline session across all test days.

In this study we used a unique design, which involved two test days for the ADHD group (one treatment-free cognitive session in each day, followed by a placebo/MPH treatment and a second cognitive session) and one test day for the controls group (two treatment-free cognitive sessions separated by an hour break). This design enabled us to investigate the differences in ERPs for ADHD vs. Controls, MPH vs. placebo, and treatment- vs. treatment-free sessions. Furthermore, the design allowed us to account for possible learning and fatigue effects. We also examined the data over a wide time window (0-1000ms post stimulus), in search of possible differences in later components.

When investigating differences between the first, pre-treatment session of ADHD (S1) and the first session of the controls (H1), significant differences were found in frontal regions only in the LC (650-800ms), which was lower for ADHD compared to controls (Fig 1). Late ERP components are thought to represent post-action processes, such as updating of working memory or retrieval from long term memory [13] and were found to be affected by the difficulty of the task [53]. The differences we found between ADHD and controls in this late component could therefore result from the ADHD subjects’ difficulty to quickly suppress the previous stimulus, maintain working memory demands, and prepare themselves for the upcoming event. No amplitude differences between the S1 and H1 conditions were found in earlier components. This result is consistent with Sunohara et al., 1999 [32], but not with several other studies [18,19,54]. However, this could be attributed to the different design used in those studies, which involved a different CPT task and an examination of ≤19 electrodes.

To assess the involvement of learning effects, we tested the treatment-free sessions of the ADHD subjects for “Session” effects. As we hypothesized, our results indicate that no learning effects were evident across the two test days. In addition, no effect of “Order” (whether MPH was administrated in the first or second day) was evident. This confirms our ability to disregard the day label and group the conditions such that they reflect only the type of treatment (“pre-PLB”, “pre-MPH”, “post-PLB” and “post-MPH”).

In agreement with our hypothesis, studying the effects of MPH on ERPs revealed higher P3 amplitude following MPH compared to placebo in parietal and frontal electrodes in the ADHD group (Fig 2). However, while we expected MPH effects on the P3 latency, none were found. The P3 component is often attributed to attention allocation and updating [12]. Differences in the amplitude of this component could therefore be interpreted as improved attention of the ADHD subjects after MPH ingestion compared to placebo. Several other studies also found P3 amplitude differences between MPH and placebo [27–30], yet Sunohara et al., 1999 [32] only found latency-related differences. The authors claimed that the absence of amplitude differences between the MPH and placebo conditions was most likely attributed to the averaging techniques employed, as amplitude differences were found when the ERPs were averaged only based on stimulus category (the targets (in their case, a repeated-letter) and non-targets), rather than on both stimulus and response categories (correct or incorrect responses). However, our ERPs were averaged over both stimulus and response category (Go condition *and* correct responses), and differences in the P3 amplitude were still evident. We therefore hypothesize that the inconsistency in the results, despite relative similarity in the design, could be attributed to their different conditions for placebo administration. In our design, the placebo was given only in the afternoon, after the first cognitive task was performed. In Sunohara et al., 1999 [32] placebo was given on three possible occasions (afternoon of the first day and morning/afternoon of the second day), and not necessarily after a cognitive task was already performed.

While several studies [27,28,32,34] found effects of MPH on amplitudes or latencies of earlier components such as N1, N2 and P2, our analysis did not reveal any difference between placebo and MPH in those components. However, these studies used cognitive tasks such as CPT or auditory oddball tasks, while we used the SART Go/No-Go task. Since early components are thought to reflect feature detection and early decision making [55], the inconsistencies between the studies could result from different tasks which recruit different aspects of decision making. Here we focused on the correct Go responses, which require the ability to maintain sustained attention but not response inhibition, and this may account for the lack of changes in the earlier components. When comparing our results only to studies which used the Go/No-Go or the similar Stop Signal task, inconsistent results regarding the Go condition arise: some found differences in early components only for the No-Go condition but not for the Go condition [56–58], while others found differences for both Go and No-Go conditions [21,59,60].

Since the P3 component differed between placebo vs. MPH conditions, we examined whether the P3 amplitude correlated to behavioral indices. We focused on the RTSD (standard deviation of the response time) of the ADHD subjects, which was found to be significantly lower after MPH compared to placebo, in consistency with the literature [48,61]. Interestingly, we found correlations between the P3 amplitude in the post-placebo session and the RTSD of the ADHD subjects (Fig 5). Specifically, the post-placebo P3 amplitude was found to be negatively correlated with the RTSD, linking lower amplitude to higher RTSD. Since higher RTSD was previously shown to indicate attention deficit [62], we infer that *lower* post-placebo P3 amplitude indicates *lower* levels of attention capabilities. This conclusion is consistent with our findings that MPH intake resulted in *lower* RTSD (hence in *higher* level of attention), as well as *higher* P3 amplitude compared to placebo.

To account for within-subject variations that might be caused by time-on-task effects and by mental fatigue, our design included two pre-treatment sessions, prior to both placebo and MPH administration. Interestingly, we found differences in the P3 amplitude when comparing between the pre-placebo and the post-placebo conditions, but not between the pre-MPH and post-MPH conditions. The lack of differences between pre- and post-MPH sessions could result from an inhibiting effect of the MPH, as suggested by Nigg, 2001 [63]. It has been previously shown that time-on-task causes mental fatigue and decreases the amplitude of the P3 component [38,41,64]. It has also been shown that time-on-task had a greater effect on ADHD subjects compared to healthy controls and resulted in a decrease of their attention capabilities [65,66]. The decrease we found in the P3 amplitude of the post-placebo condition compared to the pre-placebo condition could therefore be the result of time-on-task and increased fatigue. As MPH is suggested to improve response inhibition [67], we hypothesize that the decrease in the P3 amplitude in the post-placebo condition is inhibited by the MPH, and thus there is no evident difference when comparing the pre-MPH and post-MPH conditions. This hypothesis is strengthened by the correspondence of the ΔControl waveform (the difference between the two control sessions) and the ΔMPH waveform (the difference between the post-MPH and the pre-MPH sessions) (Fig 3), suggesting that the MPH normalizes the waveform of the ADHD subjects in the direction of the healthy controls’ waveform. Furthermore, the increase of RTSD in post-placebo compared to pre-placebo, and its decrease post-MPH, is another indication of possible fatigue and time-on-task effects which are inhibited by MPH intake [41].

Although we used a more optimal design than most previous studies, our study has limitations that need to be considered. Due to the limited number of participants, we did not use a narrow age band, and therefore could not account for age variation. However, our design was more focused on examining the repeatability within-subjects, and therefore age was less of a concern. For the same reason, we could not account for the different ADHD sub-types. In order to account for these limitations in retrospect, we included the age and sub-type as covariates in the statistics and found no significant relations. While this limitation does not appear as a substantial one in our case, future improvement of our design should take these factors into account.

In conclusion, this study tested differences between adolescents with ADHD and controls during attentional effort and investigated the normalizing effects of MPH while accounting for effects of placebo, learning and fatigue. We found MPH to have a normalizing effect on the P3 component, which decreased following placebo. This effect was evident due to our unique design, which included a treatment-free baseline session before each placebo or MPH treatment. In accordance to our hypothesis, we did not find between-days learning effects, yet we identified effects between consecutive sessions which could be attributed to task-on-time/fatigue. This result emphasizes the need for well-designed within-subject studies which account for between-days variability, especially in the context of the literature inconsistencies regarding ADHD. The differences we found between ADHD and controls in the late component suggest that the attention difficulties associated with the disorder are also manifested during the preparation for the next task. This highlights the importance of broader outlook on the ERP waveform to later times than usually considered in the context of ADHD.

## Supporting information

S1 Fig Schematic illustration of the division of the electrodes into groups [52]. The electrodes are placed according to the 10/20 International system. PFC = Prefrontal Cortex; SM = Sensory-motor; Par = Parietal; Occ = Occipital; Temp = Temporal.
(DOCX)
