# Supplementary material for: Effects of methylphenidate on the ERP amplitude in youth with ADHD: A double-blind placebo-controlled cross-over EEG study
Source: PLoS One. 2019 May 31;14(5):e0217383. doi: 10.1371/journal.pone.0217383 (PMC6544236; doi:10.1371/journal.pone.0217383)
Supplement: S1 Fig — The electrodes are placed according to the 10/20 International system. PFC = Prefrontal Cortex; SM = Sensory-motor; Par = Parietal; Occ = Occipital; Temp = Temporal. (DOCX) [file pone.0217383.s001.docx]

**Supplementary**

PO8

PO4

PO3

PO7

F2

F4

F6

F8

FC2

FC5

FC1

F3

F7

F5

FP1

FT7

T7

TP7

TP8

T8

FT8

POz

Pz

CPz

Iz

Oz

O2

O1

AF4

FP2

AF8

FC6

FC4

AF7

CP1

CP3

P10

P8

P6

P4

P2

CP6

CP4

CP2

P1

P3

P5

P7

P9

CP5

C6

C4

C2

Cz

C1

C3

C5

FC3

F1

AF3

FCz

Fz

AFz

FPz

**Par**

**Mid**

**Mid SM**

**Occ**

**Mid**

**Temp**

**Right SM**

**Left SM**

**Right PFC**

**Left PFC**

**PFC**

**Right**

**Left**

**Temp**

**Left Par**

**Right Par**

**Right Occ**

**Left Occ**

**Mid**

Figure S1. Schematic illustration of the division of the electrodes into groups (1). The electrodes are placed according to the 10/20 International system. PFC = Prefrontal Cortex; SM = Sensory-motor; Par= Parietal; Occ = Occipital; Temp = Temporal.

1. Naim-Feil J, Rubinson M, Freche D, Grinshpoon A, Peled A, Moses E, et al. Altered brain network dynamics in schizophrenia: A cognitive electroencephalography study. Biol Psychiatry Cogn Neurosci Neuroimaging. 2018;3(1):88–98.
